# Morphine? No! No! No!

**Published:** 1890-02

**Authors:** M. A. A. Wolff

**Affiliations:** Gainesville, Texas


### MORPHINE? NO! NO! NO!

M. A. A. Wolff, M. D., Gainesville, Texas.

January 11th, prescribed for fourteen days old baby. January 13th, for the mother. They had been sick for eight to ten days, during which time she had been regaled with plenty of Quinine *et id omne,* getting worse all the time. The indicated remedy, one dose, cured as far as I knew then, but on the 15th I was recalled. I found her to all purpose cured, no headache, no deafness, etc. Well, they called me on account of a lump that had been felt in her left breast, near sternum. She had it there for a good while. Could I cure that as the rest? Phytolacca, as long as I have known its use, had never failed, so this was my prescription. After twenty-four hours there was a softening, so went on with it till I returned from a trip—the 20th. It was softening, but an abscess forming; on account of color, pains, etc., prescribed Bell.$^{30}$ On the 24th I found her in bed, the pains had returned and she could not be up; Bell.$^{200}$. In the evening her husband came to the office. Her pains were so excruciating and distressing, she wanted Morphine. No! No! No! I do not give Morphine, but wait a little. I looked the case up, gave him one powder Hepar-sul.$^{1200}$ (the highest I had). Next day I found her up. The pains were soon relieved, and under the circumstances she felt comfortable. Abscess progressed, opened, and she is all right.

